# UV-LED as a New Emerging Tool for Curable Polyurethane Acrylate Hydrophobic Coating

**DOI:** 10.3390/polym13040487

**Published:** 2021-02-04

**Authors:** Siti Khairunisah Ghazali, Nadia Adrus, Rohah A. Majid, Fathilah Ali, Jamarosliza Jamaluddin

**Affiliations:** 1 School of Chemical and Energy Engineering, Faculty of Engineering, Universiti Teknologi Malaysia, Johor, Skudai 81310, Malaysia; skhairunisah2@graduate.utm.my (S.K.G.); nadia@utm.my (N.A.); r-rohah@utm.my (R.A.M.); 2 Department of Biotechnology Engineering, Faculty of Engineering, International Islamic University Malaysia, Kuala Lumpur, Gombak 53100, Malaysia; fathilah@iium.edu.my

**Keywords:** UV-LED, fluorinated polyurethane acrylate, surface properties

## Abstract

The elimination of mercury, low energy consumption, and low heat make the ultraviolet light-emitting diode (UV-LED) system emerge as a promising alternative to conventional UV-mercury radiation coating. Hence, a series of hydrophobic coatings based on urethane acrylate oligomer and fluorinated monomer via UV-LED photopolymerisation was designed in this paper. The presence of fluorine component at 1160 cm^−1^, 1235 cm^−1^, and 1296 cm^−1^ was confirmed by Fourier Transform Infra-Red spectroscopy. A considerably high degree C=C conversion (96–98%) and gel fraction (95–93%) verified the application of UV-LED as a new technique in radiation coating. It is well-accepted that fluorinated monomer can change the surface wettability as the water contact angle of the coating evolved from 88.4° to 121.2°, which, in turn, reduced its surface free energy by 70.5%. Hence, the hydrophobicity of the coating was governed by the migration of the fluorine component to the coating surface as validated by scanning electron and atomic force microscopies. However, above 4 phr of fluorinated monomer, the transparency of the cured coating examined by UV-visible spectroscopy experienced approximately a 16% reduction. In summary, the utilisation of UV-LED was a great initiative to develop green aspect in photopolymerisation, particularly in coating technology.

## 1. Introduction

Hydrophobicity is one of the surface properties that has been enthusiastically explored by researchers [1,2,3,4,5,6,7,8,9,10]. With the water contact angle exceeding 90°, the hydrophobic surface is not only water-repelling but also delivers captivating uses such as anti-fouling, anti-icing, anti-corrosion, and self-cleaning effects [11,12,13]. This appealing feature has been a matter of interest in various sectors, including automotive, textile industry, and optical devices. For instance, Suradi et al. effectively prepared a PET textile with switchable ability from a hydrophilic to hydrophobic surface [14].

The presence of low surface free energy materials is one of the predominant strategies for manipulating surface properties. For this idea, fluorinated monomers or polymers have been employed due to their exceptionally low surface free energy. It has also been well-recognized that fluorinated polymer has outstanding and distinctive properties due to the unique nature of fluorine atom. Their uniqueness includes high thermal, chemical, and weather resistance, low refractive index, low dielectric constant, and high inertness towards moisture adsorption, as well as interesting water and oil repellence [15,16,17,18,19,20,21]. Due to these reasons, fluorinated materials can be applied in wide range of applications such as chemical resistance coating, non-corrosive materials, anti-fouling coating, and as an interlayer dielectric [15,16,17,19].

In order to implement a safer and more environmentally friendly procedure for the hydrophobic coating, UV curing was adopted. UV curing consumed less energy, as it exhibited faster curing rates compared to traditional thermal curing [22]. On top of that, UV curing enables the establishment of solvent-free coating formulation without the exposure to the volatile organic compounds (VOCs). With that respect, UV curing provides an alternative solution for coating industries.

To fulfil the requirement of coating formulation, three basic components consisting of oligomer, monomers, and photoinitiator are selected in order to achieve the desired properties of final products. Dominantly, acrylate-functionalised oligomer and monomers are frequently used due to its higher reactivity [23]. Urethane acrylate oligomer with outstanding properties such as high thermal stability, flexibility, high abrasion resistance, and improved weather resistance [24,25] is one of the popular materials of choice for the UV curing system.

In the recent years, UV-LED light source has progressively emerged in various fields such as ink and printing, stereolithography, adhesive, hydrogel and wood coating [26,27,28,29,30]. This is because UV-LED consumes 50–75% less energy than the equivalent mercury arc lamp and has narrow wavelength distribution [27,28,29]. The capability to operate promptly makes the UV-LED system superior to the existing UV mercury especially in term of faster curing. In addition, UV LED is suitable for curing heat-sensitive materials because of lower heat generation. Meanwhile, from the environmental point of view, UV-LED system is safer due to the abolition of unsafe mercury and extraction of ozone. Ultimately, UV-LED system has combated several drawbacks related to conventional UV-mercury.

At present, there has been limited work on polyurethane acrylate coating using UV-LED since most of them employed the UV-mercury curing system [15,31,32,33,34,35,36,37]. Strongone et al. [38] and Malluceli [39] reported the possibility to prepare a UV-LED curable nanocoating based on epoxy acrylate with enhanced thermal and mechanical properties. In our recent paper, the effects of UV-LED irradiation time towards curing behaviour of urethane acrylate coating was investigated. We had successfully demonstrated that the properties of coating using UV-LED was superior to the conventional UV-mercury lamp at a faster curing rate [40]. Following this, the effect of fluorinated monomer content to obtain a hydrophobic coating by utilising UV-LED technology was further explored. This is accomplished by synergising a urethane diacrylate oligomer and fluorinated monomer. We also scrutinised the curing behaviour such as degree of conversion and gel fraction, using UV-LED with respect to the surface properties of the coating. Ultimately, this study revealed that UV-LED has emerged as a useful tool with the green aspects of photopolymerisation for producing advanced properties of polymeric coating materials.

## 2. Materials and Methods

**Figure 1:**
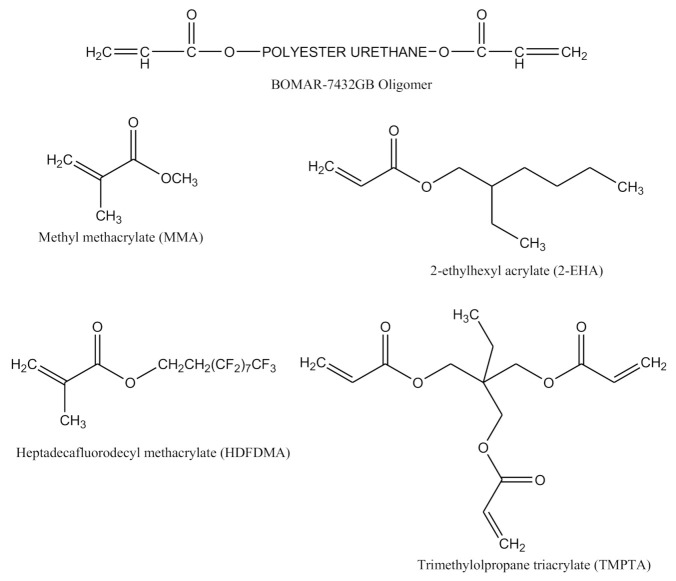
Molecular structure of the oligomer and monomers used.

In this study, urethane diacrylate oligomer (BOMAR™ 7432-GB) was generously supplied by Dymax Corporation Solutions while 2-ethylhexyl acrylate (2-EHA), methyl methacrylate (MMA), trimethylolpropane triacrylate (TMPTA) and heptadecafluorodecyl methacrylate (HDFDMA, 97%) were all obtained from Sigma Aldrich, Sdn. Bhd., Kuala Lumpur, Malaysia. The photoinitiator, Chivacure^®^ 300 was obtained from Chitec Technology Co., Ltd., Taiwan. All the chemicals were used without further purification and their structures were shown in Figure 1.

### 2.1. Preparation of UV-LED Polyurethane Acrylate

Table 1 lists the sample designation and the formulation of the prepared coating. The coating mixture was prepared by dissolving photoinitiator in the reactive monomers (MMA, 2-EHA, HDFDMA, and TMPTA) for one hour at room temperature. Then, it was added dropwise into a beaker containing oligomer and was stirred for another one hour. The prepared mixture was coated onto a clean glass substrate (10 cm × 5 cm × 0.3 cm) with the thickness of 50 μm using a bar applicator. The wet coating was exposed to the UV irradiation in Hönle UV-LED chamber with 15 cm from the light source.

### 2.2. Characterisations of the UV-LED Polyurethane Acrylate

To analyse the chemical changes in the sample, attenuated total reflectance mode Fourier Transform Infra-Red (ATR-FTIR) (Shimadzu, IR Tracer 100) was used. The ATR-FTIR spectra were obtained in the 4 cm^−1^ resolution mode with 32 scans averaged for each sample between 4000 cm^−1^ and 400 cm^−1^ at room temperature.

The degree of C=C conversion was calculated according to Equation (1):

Degree of conversion (%) = [(A_0_−A_t_)/A_0_] × 100 (1)

where A_0_, is the peak area of 802–817 cm^−1^, including the absorption peak at 810 cm^−1^ before the UV irradiation and A_t_ is the peak area at UV irradiation time, t.

Gel fraction measurement was determined by soaking the sample in toluene for one day at room temperature. The UV-curable film was filtered and dried at 50 ℃ until its weight was constant. The gel fraction of the UV-cured film was then calculated as Equation (2):

Gel fraction (%) = (w_1_/w_0_) × 100 (2)

where w_1_, is the residual weight of the cured sample [weight after filtration and drying (g) and w_0_ is the weight of the cured sample (g).

Surface wettability of the coating was quantifying based on contact angle measurements. The water and di-iodomethane static contact angle were performed using optical contact angle measurement system (CAM 101, KSV Instruments) by the sessile drop technique at room temperature. For each sample, at least five measurements were recorded at different parts of the coating surface. The surface free energy of the coating was determined using Fowkes method as in Equations (3) [41].

(3) $$\gamma_{s}=\gamma_{s}^{d}+\gamma_{s}^{p}$$

where $\gamma_{s}$ is the surface free energy of solid surface, $\gamma_{s}^{d}$ is the surface free energy of dispersive component (DIM) and $\gamma_{s}^{p}$ is the surface free energy of polar component (water). Equations (4) and (5) described the calculation of the surface free energy of each component based on their respective contact angle, θ and surface tension of the measuring liquid, $\gamma_{l}$ as stated in Equation (6).

(4) $$\gamma_{s}^{d}=0.25\gamma_{l}{\left(1+\cos\theta \right)}^{2}$$

(5) $$\gamma_{s}^{p}=\;\frac{{\left[0.5\gamma_{l}\left(1+\cos\theta \right)-{\left(\gamma_{s}^{d}\gamma_{l}^{d}\right)}^{0.5}\right]}^{2}}{\gamma_{l}^{p}}$$

(6) $$\gamma_{l}=\gamma_{l}^{d}+\gamma_{l}^{p}$$

To apply Fowkes method in calculating surface free energy of a solid, the contact angle of the solid must be first measured using two different measuring liquids namely polar liquid and dispersion liquid. Usually water and di-iodomethane (DIM) are used as the measuring liquids. Water is considered as dominant polar component liquid as its $\gamma_{l}^{p}$ = 51.0 mJ/m^2^ and $\gamma_{l}^{d}$ = 21.8 mJ/m^2^. Meanwhile, DIM acted as the dispersive component, where $\gamma_{l}=\gamma_{l}^{d}$ = 50.8 mJ/m^2^.

Surface morphology of the coating was analysed using Scanning Electron Microscopy (SEM) (JEOL, JSM-6390LV) under an acceleration voltage of 15 kV. The samples were sputtered with a thin layer of gold prior to testing.

Atomic Force Microscopy (AFM) was performed with tapping mode (JPK Instrument) under ambient conditions. In brief, the silicon tip was scanned in a 5 µm × 5 µm areas across the sample surface. The coating surface roughness for each sample was evaluated based on the AFM height image.

The UV-Visible spectrum was measured using a UV-visible spectrophotometer (UV-1800, Shimadzu). The spectrum was recorded at 800–300 nm and the transmittance at 550 nm was highlighted as the indicator of the coating transparency.

## 3. Results and Discussion

### 3.1. Chemical Analysis and Curing Behaviour

The fluorinated polyurethane acrylate or FPUA coating was prepared with different content of HDFDMA at ambient temperature (25–27 °C). The dilution capacity or the solubility threshold of the mixture decreased as the HDFDMA content increased, which consequently led to poor miscibility (heterogeneous mixture). According to Park et al. [36], the low surface tension of the fluorinated acrylate component consisting of hydrophobic and oleophobic perfluoroalkyl moieties was one of the factors that trigger to this case. In addition, the poor compatibility of the HDFDMA and the urethane acrylate oligomer was also caused by their dissimilar property behaviour (hydrophobic and hydrophilic state). In this study, the mixture containing 0–10 phr of HDFDMA apparently had a homogenous system and the mixture started to become heterogeneous when HDFDMA was further added up to 12 phr.

**Figure 2:**
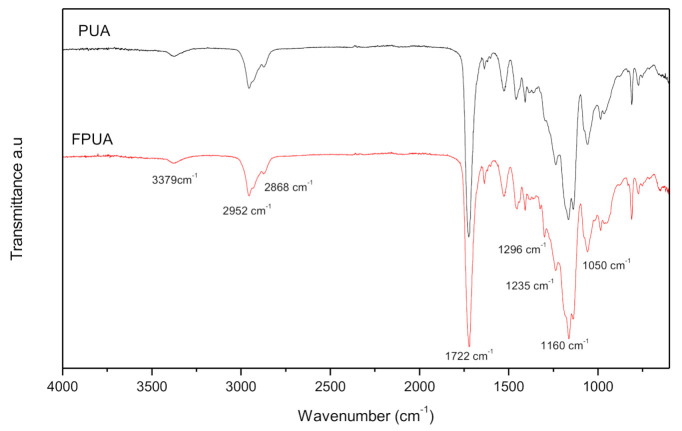
FTIR spectra of the pristine polyurethane acrylate and fluorinated polyurethane acrylate, FPUA-4.

The structures of the prepared FPUA coating were confirmed by ATR-FTIR analysis. Figure 2 depicts the spectra of pristine PUA and FPUA. It was found that in both spectra, there was a characteristic peak of N-H at 3379 cm^−1^ and C-H aliphatic stretches bands at 2952 cm^−1^ (methyl group, -CH_3_) and 2868 cm^−1^ (methylene group, -CH_2_) were also observed. The strong absorption around 1722 cm^−1^ in the spectra corresponded to the vibration of C=O group. The absorption band at 1050 cm^−1^ indicates C-O-C in the formulation. Besides that, the stretching vibrations of -CF_2_ group at 1160 cm^−1^, -CF_3_ group at 1235 cm^−1^, 1296 cm^−1^ were all detected in the FPUA coating [9,19,36].

On the other hand, the degree of C=C conversion and gel fraction were also calculated for the fluorinated urethane coating, and the results are presented in Table 2. Above 6 phr of HDFDMA, there is a slight change in conversion, approximately 1–2% of reduction. This reduction can be due to the bulky structure of the HDFDMA, that might be hindered the reaction of the double bond conversion in the system. This considerably high C=C conversion exceeding 95% evidenced the application of the UV-LED curing as a new promising alternative in coating.

A similar trend was also observed in gel fraction, where the values decreased with the increasing of HDFDMA content, and it reflects the degree of C=C conversion. However, the UV-cured coating still exhibits a high degree of cross-linking (above 90%). Çanak et al. [17] also reported similar findings where the gel fraction of the conventional UV-curable fluorinated acrylate film almost plateau even the fluorinated monomer content added was up to 30 wt %. From this, it can be postulated that the fluorinated monomer content did not significantly affect both degree of C=C conversion and gel fraction of the cured coating.

### 3.2. Contact Angle and Surface Free Energy

**Figure 3:**
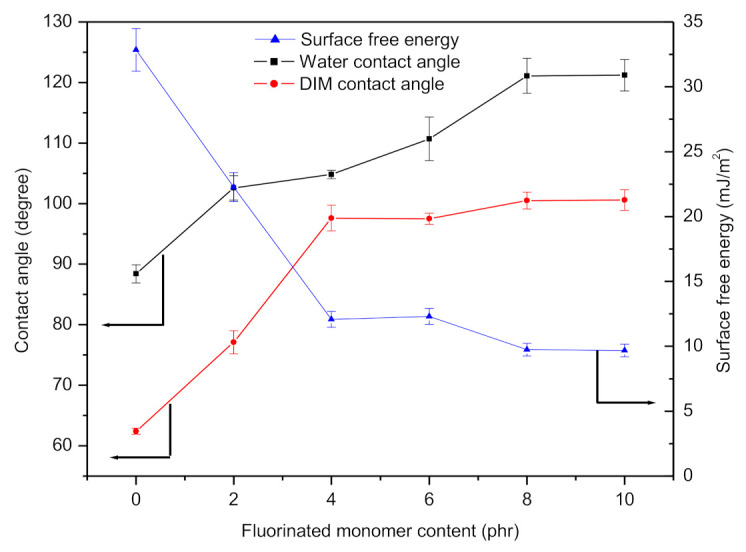
Contact angle (°) of the coating and their respective surface free energy (mJ/m^2^) at different fluorinated monomer content (phr).

**Figure 4:**
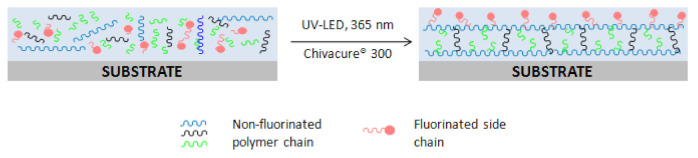
Illustration of the possible migration of fluorinated component in the system upon UV-LED irradiation.

The contact angle is the angle at which a liquid/vapour interface meets the solid surface and is determined by the interactions across the three interfaces—solid/liquid, solid/gas, and liquid/gas [42]. Hydrophobicity or hydrophilicity of the surface was quantified by the measurement of water contact angle (WCA) where WCA less than 90° was considered as hydrophilic surface (good wetting behaviour) while WCA more than 90° was described as hydrophobic surface (poor wetting behaviour). In this study, the effect of HDFDMA content on the surface wettability of the cured coating was investigated. Basically, incorporation of fluorinated moieties into polymer film has been proved to be effective in reducing surface free energy [43,44]. Initially, the contact angles of PUA for both water and DIM were 88.4° and 62.4°, respectively. However, when the HDFDMA was added, the contact angles for both measuring liquid started to increase steadily and in fact, the values continuously increased with the increasing content of fluorinated monomer as shown in Figure 3. The hydrophobic surface, which is represented by WCA reaching its highest (121°) when the cured coating containing more than 8 phr of HDFDMA. The increment in contact angle for this coating was related to the enrichment of fluorinated structure at the coating surface or polymer surface, as illustrated in Figure 4 [9,21,43]. At higher HDFDMA content, the tendency of contact angle to increase became plateau inferring to the fluorine enrichment has reached its maximal value. This can be a plausible explanation for the trend showed at 8 phr and 10 phr of HDFDMA content.

For surface free energy analysis, Fowkes derivations were employed. The data obtained are presented in Figure 3 as well. The surface free energy of UV-cured coating was decreased from 32.85–9.68 mJ/m^2^ when HDFDMA was added. The changes of surface free energy were due to the migration of fluorinated moieties towards the coating surface. The lower surface tension approximately 11 mJ/m^2^, possessed by the fluorinated monomer than the polymeric backbone (urethane), caused the fluorine structure to segregate and dominated the surface in order to reduce the overall surface tension of the system [21]. Therefore, lower surface energy was achieved when the surface was uniformly covered by the fluorinated arrays.

### 3.3. Surface Morphology

**Figure 5:**
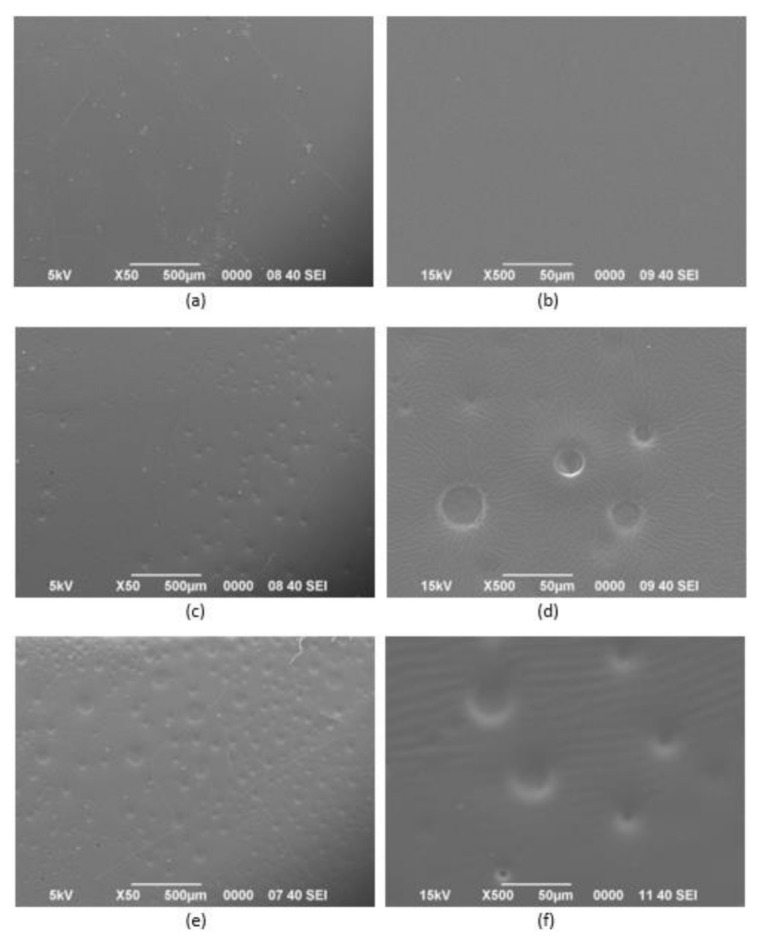
SEM images of the pristine polyurethane acrylate and fluorinated polyurethane acrylate: (**a**) PUA at 50 magnification; (**b**) PUA at 500 magnification; (**c**) FPUA-4 at 50 magnification; (**d**) FPUA-4 at 500 magnification; (**e**) FPUA-10 at 50 magnification and (**f**) FPUA-10 at 500 magnification.

SEM was used in this work to examine the surface morphology of the polymer film and Figure 5 shows the respective SEM images. It can be noted that the PUA film Figure 5a,b appeared to have a smooth surface compared to FPUA as shown in Figure 5c–f. According to the presented figures, FPUA-10 film had more salient features on the surface than FPUA-4 which exhibited some smooth regions (Figure 5c) resulting from the enrichment of fluorinated group on the surface during the UV curing. Microphase separation was also known as the factor to trigger such surface [45,46]. The content of dissimilar components of urethane acrylate and fluorinated methacrylate monomer, by which drive the phase segregation thermodynamically, was an important parameter for tuning the bulk and the surface features [46].

As demonstrated earlier, the SEM images were in agreement with the measurement of contact angle as higher WCAs were accomplished when greater amounts of fluorinated monomer content were combined. It can be obviously observed that there was the enrichment of the fluorinated component at the outermost surface compared to the neat PUA.

### 3.4. Surface Roughness

**Figure 6:**
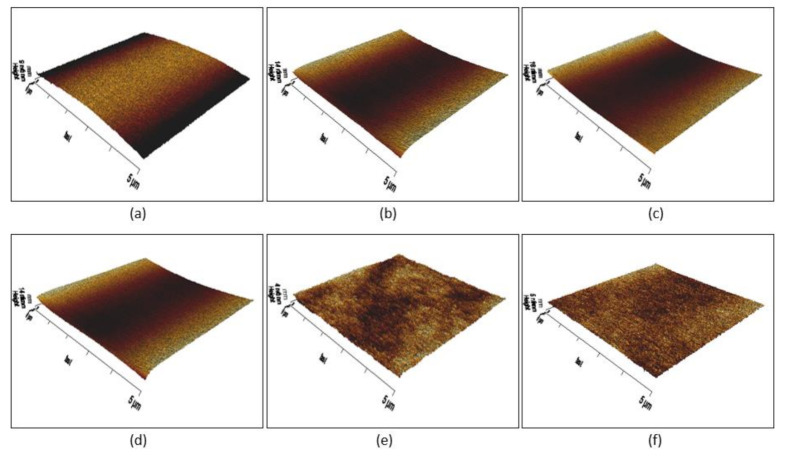
Atomic force microscopy (AFM) of the UV-LED curable polyurethane coating: (**a**) pristine PUA; (**b**) FPUA-2; (**c**) FPUA-4; (**d**) FPUA-6; (**e**) FPUA-8; and (**f**) FPUA-10.

The surface roughness plays an important role in surface properties. In the case of a hydrophobic surface, the apparent water contact angle is enhanced by its surface roughness. Hence, the surface of the coating was analysed by AFM and portrayed in Figure 6. Depending on the amount of HDFDMA, the surface roughness was significantly varied.

It can be clearly seen that the 3D image of the coating in Figure 6 displayed a transition of smooth to a rougher surface. The differences in height of the peak-to-valley (light to darker region) could be due to the emergence of the fluorinated component towards the coating surface. Since the coating surface was covered and dominated with the fluorinated segments as the HDFDMA content increased, as depicted in Figure 6e,f, the surface of the coating became more heterogeneous. As a result, more height variations can be detected and make the surface rougher. From this observation, it can be a possible reason to elucidate the change of the surface roughness of the coating.

**Figure 7:**
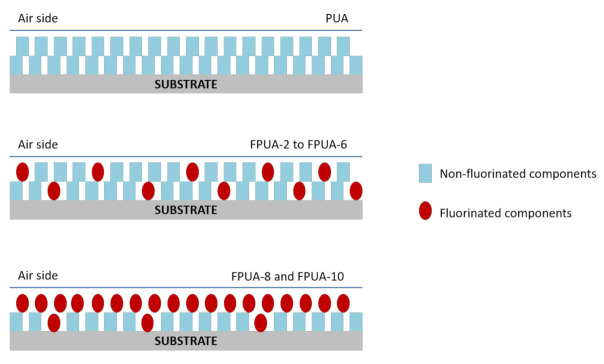
Schematic illustration of the enrichment and reconfiguration of fluorinated monomer that cause variation in surface roughness.

To further clarify the facts of the reconfiguration and accumulations of fluorinated side chains at the surface during the photopolymerisation, Figure 7 exhibits the schematic illustration of the possible non-fluorinated components and fluorinated side chains reconfiguration at the coating surface (air side surface). In the meantime, phase separation involving fluorinated and non-fluorinated components also occurred and as a result improving the roughness of the coating surface [45]. Straightforwardly, the chemistry of the fluoroalkyl group itself is superior to revolutionise the wetting behaviour of the surface (hydrophilic to hydrophobic) which is mostly attributed to its exceptionally low surface free energy property.

### 3.5. Optical Properties

**Figure 8:**
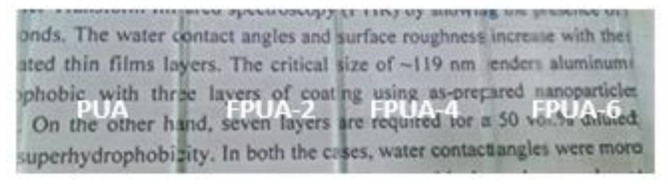
The visibility of the UV-LED curable coating at different fluorinated monomer content when placed on a piece of paper.

**Figure 9:**
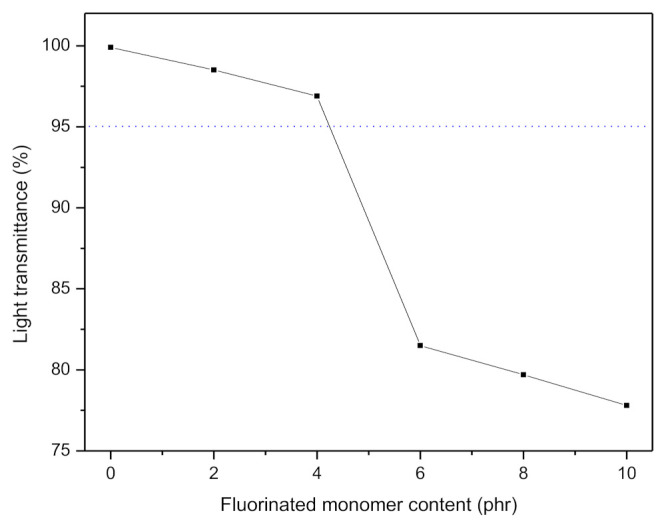
Light transmittance of the UV-LED curable coating at 550 nm.

Figure 8 shows the optical transparency of the PUA, FPUA-2, FPUA-4, and FPUA-6 coating being placed on a paper. There was no noticeable difference observed through naked eyes though. But based on Figure 9, the light transmittance was changed with the addition of HDFDMA. The light transmittance of PUA was nearly 100% (transparent) at 550 nm and gradually decreased as the HDFDMA added increased. The transmittance drastically drops at 6 phr (from 97% to 82%). The difference in transmittance could be due to the increase in the fluoroalkyl group and microphase separation, which follow the increasing content of HDFDMA monomer [36]. Accordingly, this situation led to poor compatibility of HDFDMA and urethane oligomer, which results in cloudy solution formation. Ganesh et al. [18] also reported that the relatively low solubility of fluorinated monomer within its medium (co-monomers and oligomer mixture) caused its segregation and thus affected the light transmittance property.

## 4. Conclusions

A hydrophobic coating was successfully prepared by using urethane acrylate oligomer and fluorinated-based monomer via UV-LED photopolymerisation. Notably, UV-LED photopolymerisation exhibits a high curing efficiency with 98% of C=C conversion and 95% of gel fraction. The coating surface also ultimately changed from hydrophilic to hydrophobic upon addition of HDFDMA, even at the lowest amount, which is at 2 phr. The incorporation of HDFDMA is crucial for enhancing the hydrophobicity of the coating as it lowered the overall surface free energy and led to a substantially higher water contact angle. Results from SEM and AFM confirmed the migration and surface reconfiguration of the fluorinated component towards the coating surface. Since the fluorinated component has enriched the coating surface, the transparency of the coating was also decreased with the increase in the HDFDMA content. However, the UV-curable FPUA still demonstrate a fair visibility, even at 82% of light transmittance, as exhibited by FPUA-6. Based on these findings, the coating may find its potential application as a water repellant coating or anti-fouling coating.

## Figures and Tables

**Table 1:** Composition of the UV-LED curable FPUA.

Sample Designation	Formulations					
	Oligomer (wt %)	Chivacure^®^ 300 (wt %)	MMA (wt %)	2-EHA (wt %)	TMPTA (wt %)	HDFDMA (phr ^1^)
PUA	70	4	13	8	5	0
FPUA-2	70	4	13	8	5	2
FPUA-4	70	4	13	8	5	4
FPUA-6	70	4	13	8	5	6
FPUA-8	70	4	13	8	5	8
FPUA-10	70	4	13	8	5	10
FPUA-12	70	4	13	8	5	12

^1^ phr: part per hundred.

**Table 2:** Degree of C=C conversion and gel fraction of the UV-LED curable coating.

Sample Designation	Observation of the Uncured Solution	Conversion (%)	Gel Fraction (%)
PUA	Homogeneous	98	95
FPUA-2	Homogeneous	98	94
FPUA-4	Homogeneous	98	94
FPUA-6	Homogeneous	98	94
FPUA-8	Homogeneous	97	94
FPUA-10	Homogeneous	96	93
FPUA-12 ^1^	Heterogeneous	-	-

^1^ FPUA-12 was excluded from further analysis and testing.

## Data Availability

The data presented in this study are available on request from the corresponding author.
